# Supplementary material for: Genome-wide profiling of soybean WRINKLED1 transcription factor binding sites provides insight into seed storage lipid biosynthesis
Source: Proc Natl Acad Sci U S A. 2024 Oct 30;121(45):e2415224121. doi: 10.1073/pnas.2415224121 (PMC11551420; doi:10.1073/pnas.2415224121)
Supplement: Supplementary file 1 — Appendix 01 (PDF) [file pnas.2415224121.sapp.pdf]

PNAS

Supporting Information for
Genome-Wide Profiling of Soybean WRINKLED1 Transcription Factor Binding Sites Provides Insight into Seed Storage Lipid Biosynthesis

Leonardo Jo, Julie M. Pelletier, Robert B. Goldberg, John J. Harada

John J. Harada
Email: jjharada@ucdavis.edu

Robert B. Goldberg
Email: bobg@ucla.edu

This PDF file includes:

Figures S1 to S7
Tables S1 and S2

Other supporting materials for this manuscript include the following:

Datasets S1 to S5

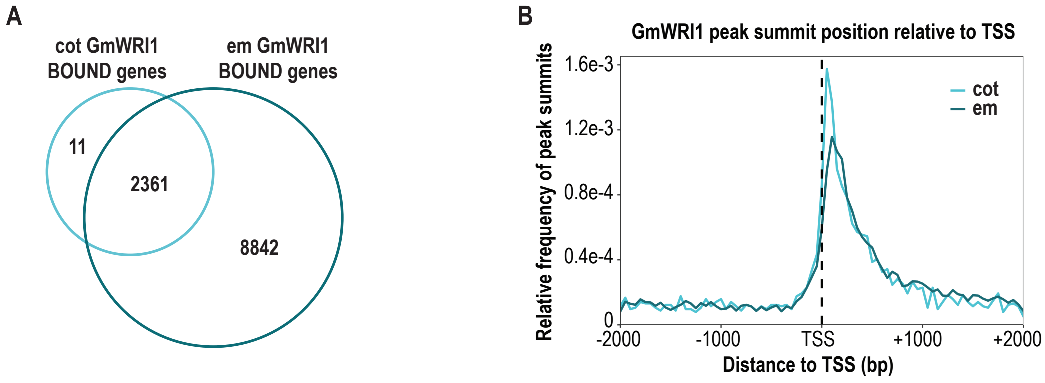

Figure S1. Comparison of GmWRI1 ChIP-Seq analyses with cotyledon and early maturation stage embryos.
(*A*) Venn diagram comparing genes bound by GmWRI1 in embryos at the cot and em stages. (*B*) Relative positions of cot and em GmWRI1 ChIP-seq peaks of bound genes relative to the TSS.

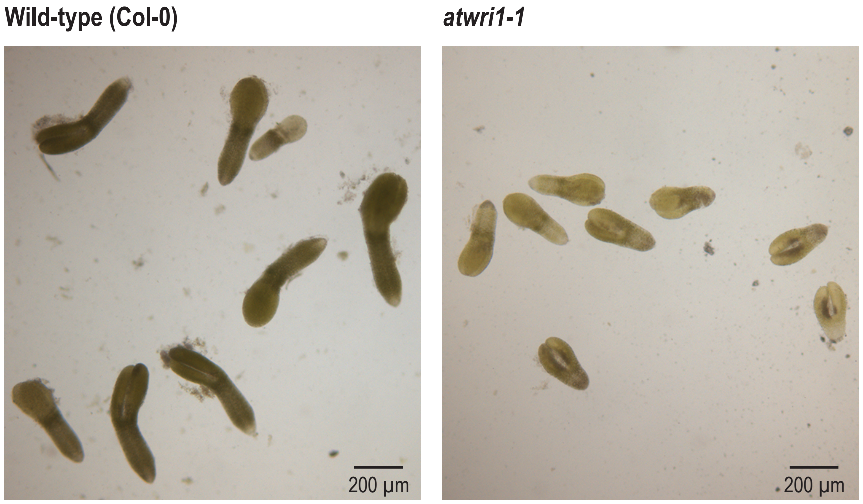

Figure S2. Comparison of whole mount 8 DAP wild-type and *atwri1* mutant embryos.

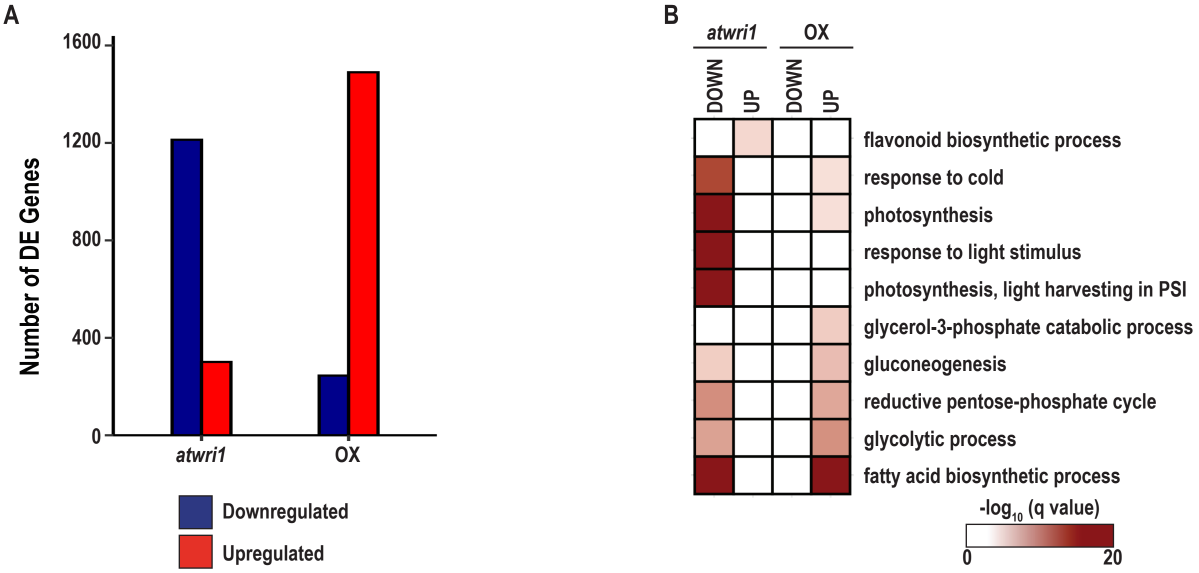

Figure S3. AtWRI1 directly regulated genes in Arabidopsis.
(*A*) Number of DE genes (two-fold, FDR < 0.05) identified in WT versus *atwri1* mutant embryos at 8 DAP and in Arabidopsis leaf cells transfected with *35S:GmWRI1* versus *35S:mCHERRY* (OX). (*B*) Heatmap shows enriched GO terms for each DE gene set (q value < 0.01).

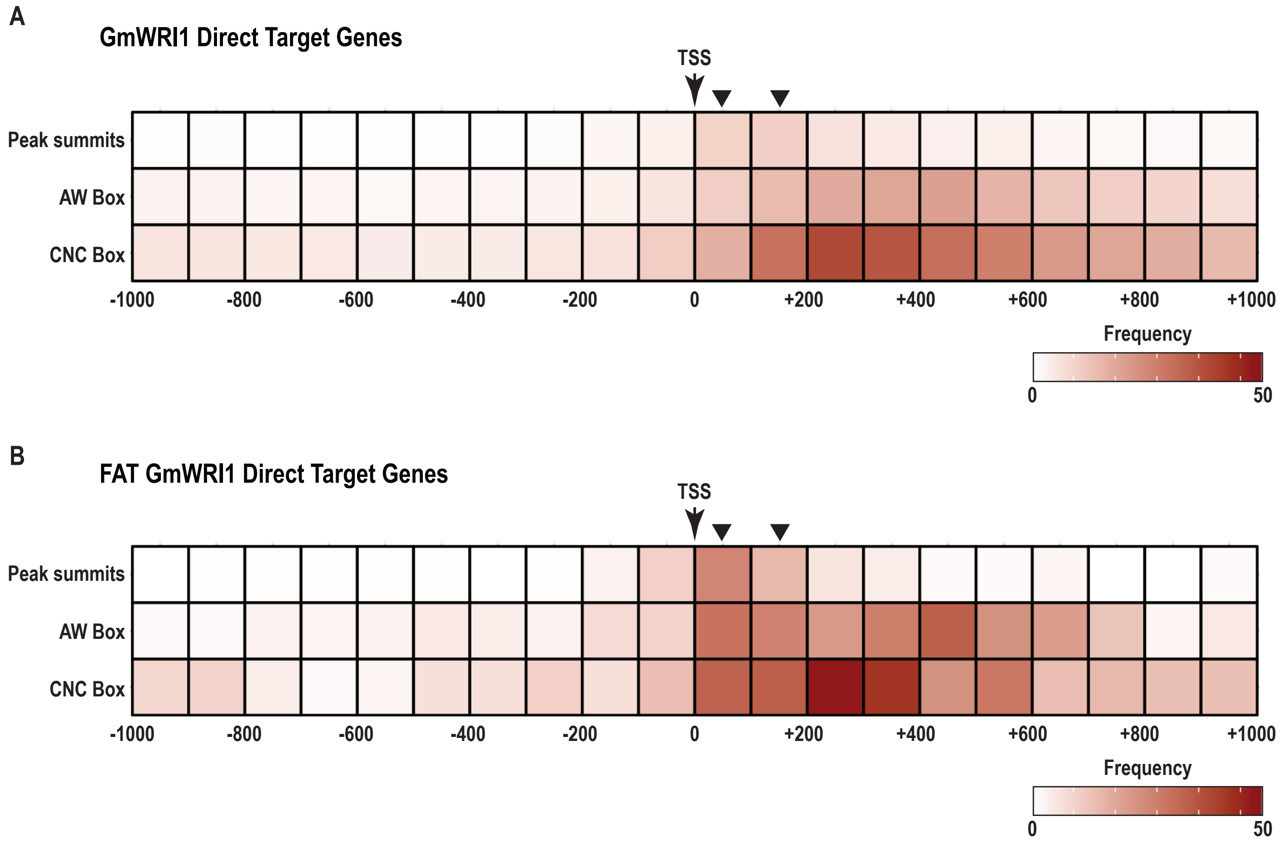

Figure S4. Positional distribution of the GmWRI1 ChIP-Seq peaks, AW Boxes, and CNC Boxes in GmWRI1 DT genes.
Heatmaps show the frequency of GmWRI1 ChIP-Seq peaks and AW and CNC Box motifs in regions 1000 bp upstream and downstream of the TSS of GmWRI1 DT (*A*) and the FAT GmWRI1 DT (*B*) genes. Arrowheads indicate regions in which ChIP-Seq peak density is highest.

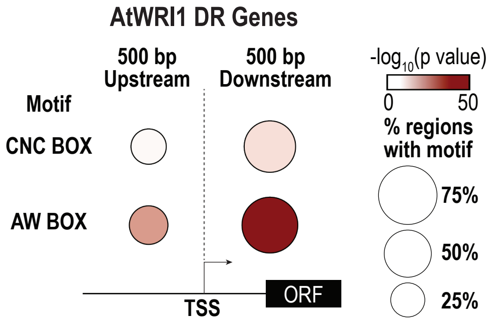

Figure S5. Enrichment of AW Box and CNC Box motifs in Arabidopsis directly regulated genes. Bubble plot showing the motif enrichment in 500 bp regions upstream and downstream the TSS of AtWRI1 DR genes. Diameter of the circles depict the frequencies at which DNA motifs were identified in the indicated regions, and the intensity of their color indicates the statistical significance of the enrichment relative to the normal distribution of a population of randomly generated regions (Bonferroni-adjusted *P* values).

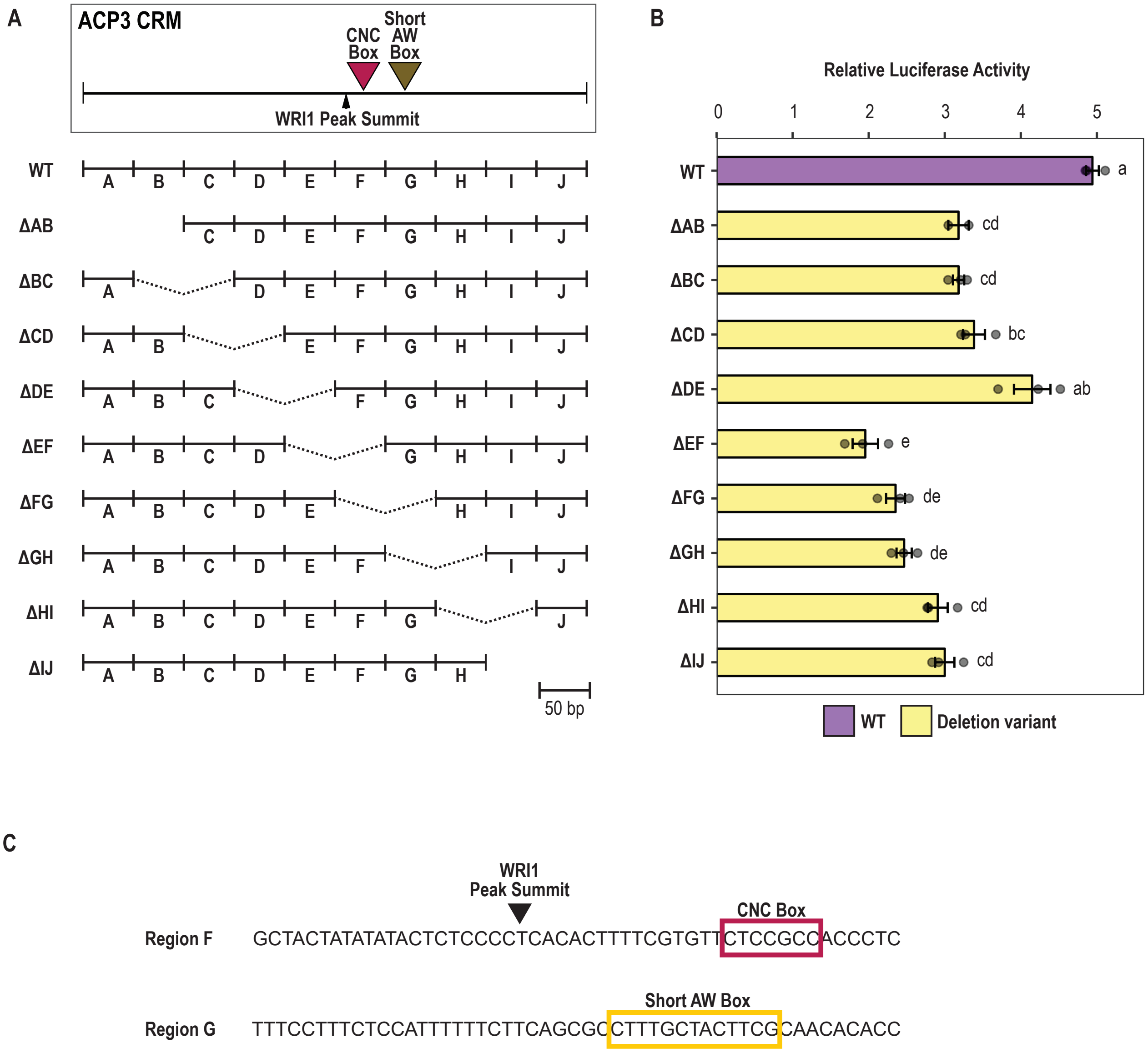

Figure S6. Functional analyses of the ACP3 binding site.
(A) Diagrams illustrating the expanded WT ACP3 binding site and deletion constructs tested. (B) Relative luciferase activity of *ACP3* WT and deletion constructs in soybean embryo protoplasts. The average firefly to Renilla luciferase activity ratio was normalized to the pDLUC15 negative control and is plotted with the standard errors (n = 3). Lower-case letters indicate the significance of comparisons based on ANOVA and post hoc Tukey test results ($P < 0.01$). (C) DNA sequence of Regions F and G, with the positions of the ChIP-Seq peak and AW and CNC Boxes indicated.

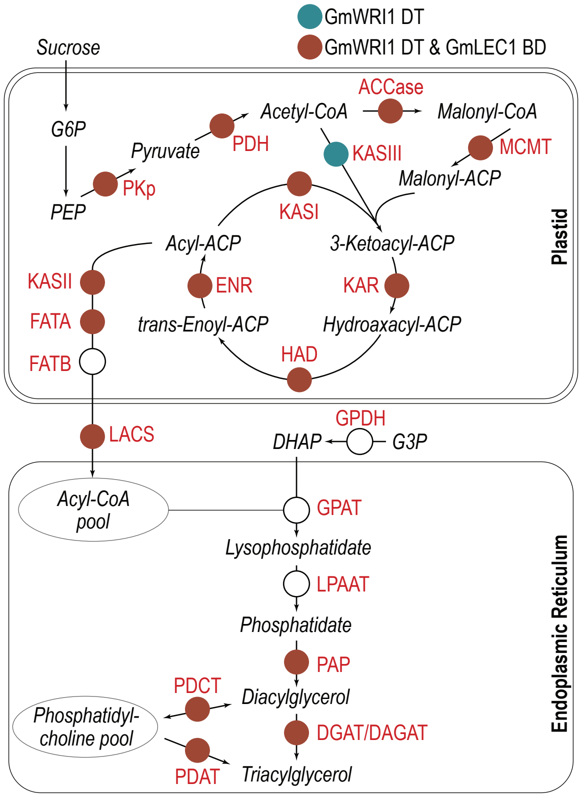

Figure S7. GmWRI1 and GmLEC1 control fatty acid and triacylglycerol biosynthesis in soybean embryos.
Simplified schematic illustrating FA and TAG biosynthetic pathways highlighting enzymes encoded by GmWRI1 DT genes that are bound (red) or not bound (teal) by GmLEC1.

Table S1. DNA Sequences for WT and Mutant Derivatives of WRI1 Binding Sites

locus	Gene name	Type	Binding site sequence
**Glyma.19G240100**	***ACP3***	WT	GATTGTGGATTTCATTATACTACTAATAAAAAATAACAATTAAAATTTACTGATAATAAAAAGTTGATCAACATGCTG<br>GTATTTTAAAATATTTTTATTTAAGAAAAAGTAGGGGTGTGAAGGAAAAATAAATAAATAAGCAAACGTCTGTAAAA<br>AGAAAGAAGCATGTGTAAAGCCCAGCCCAAAGGAAGGGGTACTTGAGATTCGGAATCCAAAGAGAGGACTCTTT<br>CCTGCAGCCAACAATCACGGGGCTACTATATATACTCTCCCCTCACACTTTTCGTGTT**CTCCGCC**ACCCTCTTTC<br>CTTTCTCCATTTTTTCTTCAGCGC**CTTTGCTACTTCG**CAACACACCACAATGGCCACCAATGCACTCGCTGGCAC<br>ATCTCTCTCCATGCGATCTCT+D4:D16CCTTCCTCAAACACAGGTATTGTTTTCCCTTCCTCTTTTCATAAATCATA
		mAW	GATTGTGGATTTCATTATACTACTAATAAAAAATAACAATTAAAATTTACTGATAATAAAAAGTTGATCAACATGCTG<br>GTATTTTAAAATATTTTTATTTAAGAAAAAGTAGGGGTGTGAAGGAAAAATAAATAAATAAGCAAACGTCTGTAAAA<br>AGAAAGAAGCATGTGTAAAGCCCAGCCCAAAGGAAGGGGTACTTGAGATTCGGAATCCAAAGAGAGGACTCTTT<br>CCTGCAGCCAACAATCACGGGGCTACTATATATACTCTCCCCTCACACTTTTCGTGTT**CTCCGCC**ACCCTCTTTC<br>CTTTCTCCATTTTTTCTTCAGCGC**aTaTaCTACTTaa**CAACACACCACAATGGCCACCAATGCACTCGCTGGCACAT<br>CTCTCTCCATGCGATCTCTCCTTCCTCAAACACAGGTATTGTTTTCCCTTCCTCTTTTCATAAATCATAACCACTAG
		mCCNCC	GATTGTGGATTTCATTATACTACTAATAAAAAATAACAATTAAAATTTACTGATAATAAAAAGTTGATCAACATGCTG<br>GTATTTTAAAATATTTTTATTTAAGAAAAAGTAGGGGTGTGAAGGAAAAATAAATAAATAAGCAAACGTCTGTAAAA<br>AGAAAGAAGCATGTGTAAAGCCCAGCCCAAAGGAAGGGGTACTTGAGATTCGGAATCCAAAGAGAGGACTCTTT<br>CCTGCAGCCAACAATCACGGGGCTACTATATATACTCTCCCCTCACACTTTTCGTGTT**aTaaGaa**ACCCTCTTTCCT<br>TTCTCCATTTTTTCTTCAGCGC**CTTTGCTACTTCG**CAACACACCACAATGGCCACCAATGCACTCGCTGGCACAT<br>CTCTCTCCATGCGATCTCTCCTTCCTCAAACACAGGTATTGTTTTCCCTTCCTCTTTTCATAAATCATAACCACTAG
		mAWmCCNCC	GATTGTGGATTTCATTATACTACTAATAAAAAATAACAATTAAAATTTACTGATAATAAAAAGTTGATCAACATGCTG<br>GTATTTTAAAATATTTTTATTTAAGAAAAAGTAGGGGTGTGAAGGAAAAATAAATAAATAAGCAAACGTCTGTAAAA<br>AGAAAGAAGCATGTGTAAAGCCCAGCCCAAAGGAAGGGGTACTTGAGATTCGGAATCCAAAGAGAGGACTCTTT<br>CCTGCAGCCAACAATCACGGGGCTACTATATATACTCTCCCCTCACACTTTTCGTGTT**aTaaGaa**ACCCTCTTTCCT<br>TTCTCCATTTTTTCTTCAGCGC**aTaTaCTACTTaa**CAACACACCACAATGGCCACCAATGCACTCGCTGGCACATCT<br>CTCTCCATGCGATCTCTCCTTCCTCAAACACAGGTATTGTTTTCCCTTCCTCTTTTCATAAATCATAACCACTAGTA
		del-AB	AAGAAAAAGTAGGGGTGTGAAGGAAAAATAAATAAATAAGCAAACGTCTGTAAAAAGAAAGAAGCATGTGTAAAG<br>CCCAGCCCAAAGGAAGGGGTACTTGAGATTCGGAATCCAAAGAGAGGACTCTTTCCTGCAGCCAACAATCACGG<br>GGCTACTATATATACTCTCCCCTCACACTTTTCGTGTT**CTCCGCC**ACCCTCTTTCCTTTCTCCATTTTTTCTTCAGC<br>GC**CTTTGCTACTTCG**CAACACACCACAATGGCCACCAATGCACTCGCTGGCACATCTCTCTCCATGCGATCTCTC<br>CTTCCTCAAACACAGGTATTGTTTTCCCTTCCTCTTTTCATAAATCATAACCACTAGTACTATGTGCTTCGCACTGC
		del-BC	GATTGTGGATTTCATTATACTACTAATAAAAAATAACAATTAAAATTTACTAAAAAGAAAGAAGCATGTGTAAAGCC<br>CAGCCCAAAGGAAGGGGTACTTGAGATTCGGAATCCAAAGAGAGGACTCTTTCCTGCAGCCAACAATCACGGGG<br>CTACTATATATACTCTCCCCTCACACTTTTCGTGTT**CTCCGCC**ACCCTCTTTCCTTTCTCCATTTTTTCTTCAGCGC<br>**CTTTGCTACTTCG**CAACACACCACAATGGCCACCAATGCACTCGCTGGCACATCTCTCTCCATGCGATCTCTCCT<br>TCCTCAAACACAGGTATTGTTTTCCCTTCCTCTTTTCATAAATCATAACCACTAGTACTATGTGCTTCGCACTGCCT
		del-CD	GATTGTGGATTTCATTATACTACTAATAAAAAATAACAATTAAAATTTACTGATAATAAAAAGTTGATCAACATGCTG<br>GTATTTTAAAATATTTTTATTTAGATTCGGAATCCAAAGAGAGGACTCTTTCCTGCAGCCAACAATCACGGGGCTA<br>CTATATATACTCTCCCCTCACACTTTTCGTGTT**CTCCGCC**ACCCTCTTTCCTTTCTCCATTTTTTCTTCAGCGC**CTT**<br>**TGCTACTTCG**CAACACACCACAATGGCCACCAATGCACTCGCTGGCACATCTCTCTCCATGCGATCTCTCCTTCC<br>TCAAACACAGGTATTGTTTTCCCTTCCTCTTTTCATAAATCATAACCACTAGTACTATGTGCTTCGCACTGCCTCTT
		del-DE	GATTGTGGATTTCATTATACTACTAATAAAAAATAACAATTAAAATTTACTGATAATAAAAAGTTGATCAACATGCTG<br>GTATTTTAAAATATTTTTATTTAAGAAAAAGTAGGGGTGTGAAGGAAAAATAAATAAATAAGCAAACGTCTGGCTAC<br>TATATATACTCTCCCCTCACACTTTTCGTGTT**CTCCGCC**ACCCTCTTTCCTTTCTCCATTTTTTCTTCAGCGC**CTTT**<br>**GCTACTTCG**CAACACACCACAATGGCCACCAATGCACTCGCTGGCACATCTCTCTCCATGCGATCTCTCCTTCCT<br>CAAACACAGGTATTGTTTTCCCTTCCTCTTTTCATAAATCATAACCACTAGTACTATGTGCTTCGCACTGCCTCTTC
		del-EF	GATTGTGGATTTCATTATACTACTAATAAAAAATAACAATTAAAATTTACTGATAATAAAAAGTTGATCAACATGCTG<br>GTATTTTAAAATATTTTTATTTAAGAAAAAGTAGGGGTGTGAAGGAAAAATAAATAAATAAGCAAACGTCTGTAAAA<br>AGAAAGAAGCATGTGTAAAGCCCAGCCCAAAGGAAGGGGTACTTGTTTCCTTTCTCCATTTTTTCTTCAGCGC**CT**<br>**TTGCTACTTCG**CAACACACCACAATGGCCACCAATGCACTCGCTGGCACATCTCTCTCCATGCGATCTCTCCTTC<br>CTCAAACACAGGTATTGTTTTCCCTTCCTCTTTTCATAAATCATAACCACTAGTACTATGTGCTTCGCACTGCCTCT
		del-FG	GATTGTGGATTTCATTATACTACTAATAAAAAATAACAATTAAAATTTACTGATAATAAAAAGTTGATCAACATGCTG<br>GTATTTTAAAATATTTTTATTTAAGAAAAAGTAGGGGTGTGAAGGAAAAATAAATAAATAAGCAAACGTCTGTAAAA<br>AGAAAGAAGCATGTGTAAAGCCCAGCCCAAAGGAAGGGGTACTTGAGATTCGGAATCCAAAGAGAGGACTCTTT<br>CCTGCAGCCAACAATCACGGGACAATGGCCACCAATGCACTCGCTGGCACATCTCTCTCCATGCGATCTCTCCT<br>TCCTCAAACACAGGTATTGTTTTCCCTTCCTCTTTTCATAAATCATAACCACTAGTACTATGTGCTTCGCACTGCCT
		del-GH	GATTGTGGATTTCATTATACTACTAATAAAAAATAACAATTAAAATTTACTGATAATAAAAAGTTGATCAACATGCTG<br>GTATTTTAAAATATTTTTATTTAAGAAAAAGTAGGGGTGTGAAGGAAAAATAAATAAATAAGCAAACGTCTGTAAAA<br>AGAAAGAAGCATGTGTAAAGCCCAGCCCAAAGGAAGGGGTACTTGAGATTCGGAATCCAAAGAGAGGACTCTTT<br>CCTGCAGCCAACAATCACGGGGCTACTATATATACTCTCCCCTCACACTTTTCGTGTT**CTCCGCC**ACCCTCCCTT<br>CCTCAAACACAGGTATTGTTTTCCCTTCCTCTTTTCATAAATCATAACCACTAGTACTATGTGCTTCGCACTGCCT
		del-HI	GATTGTGGATTTCATTATACTACTAATAAAAAATAACAATTAAAATTTACTGATAATAAAAAGTTGATCAACATGCTG<br>GTATTTTAAAATATTTTTATTTAAGAAAAAGTAGGGGTGTGAAGGAAAAATAAATAAATAAGCAAACGTCTGTAAAA<br>AGAAAGAAGCATGTGTAAAGCCCAGCCCAAAGGAAGGGGTACTTGAGATTCGGAATCCAAAGAGAGGACTCTTT<br>CCTGCAGCCAACAATCACGGGGCTACTATATATACTCTCCCCTCACACTTTTCGTGTT**CTCCGCC**ACCCTCTTTC<br>CTTTCTCCATTTTTTCTTCAGCGC**CTTTGCTACTTCG**CAACACACCACCACTAGTACTATGTGCTTCGCACTGCCT
		del-IJ	GATTGTGGATTTCATTATACTACTAATAAAAAATAACAATTAAAATTTACTGATAATAAAAAGTTGATCAACATGCTG<br>GTATTTTAAAATATTTTTATTTAAGAAAAAGTAGGGGTGTGAAGGAAAAATAAATAAATAAGCAAACGTCTGTAAAA<br>AGAAAGAAGCATGTGTAAAGCCCAGCCCAAAGGAAGGGGTACTTGAGATTCGGAATCCAAAGAGAGGACTCTTT<br>CCTGCAGCCAACAATCACGGGGCTACTATATATACTCTCCCCTCACACTTTTCGTGTT**CTCCGCC**ACCCTCTTTC<br>CTTTCTCCATTTTTTCTTCAGCGC**CTTTGCTACTTCG**CAACACACCACAATGGCCACCAATGCACTCGCTGGCAC

locus	Gene name	Type	Binding site sequence
**Glyma.15G098500**	***ACP4***	WT	CAGAACTCATATGTCAAATAAATTTTGTTGAAGTTATCATAATAATTTTATAATATAAATGTGTTAACAAACTATATCA<br>TTAATAATATTTTAAGATAATTTTTAGTTATAATTCAGTAAGATCCTCGTATTATTTATTGTAAATTAATAAGCCCTTT<br>ACATATTAAATAACTAATGGTTGTCAGGTGCACCGAACAAAGAATAAGAAGATCCAGATTCGAGGGACATGATCT<br>CGGAGAGGCAAAGCGAACAAACGGCTACTCTCCTCCCACTTGCTATCCTCGAAATTAACGAAACGTAGTTGAAG<br>AACCCACAGAATCAGAACCCCAACACTGCCTTATGCTTTCCCTATAAATACACATTGTCCCTTCCTCTCGTCATTT<br>CAAATACAAACTCACAACACACTTTGTACACTCCGTCCCTCTTCCCTGTCTCTCAAATGGCTTCCCTGACGCAAAC
		mAW	CAGAACTCATATGTCAAATAAATTTTGTTGAAGTTATCATAATAATTTTATAATATAAATGTGTTAACAAACTATATCA<br>TTAATAATATTTTAAGATAATTTTTAGTTATAATTCAGTAAGATCCTCGTATTATTTATTGTAAATTAATAAGCCCTTT<br>ACATATTAAATAACTAATGGTTGTCAGGTGCACCGAACAAAGAATAAGAAGATCCAGATTCGAGGGACATGATCT<br>CGGAGAGGCAAAGCGAACAAACGGCTACTCTCCTCCCACTTGCTATaCaCaAAATTAAaaAAACGTAGTTGAAGAA<br>CCCACAGAATCAGAACCCCAACACTGCCTTATGCTTTCCCTATAAATACACATTGTCCCTTCCTCTCGTCATTTCA<br>AATACAAACTCACAACACAaTaTaTACACTCaaTCCCTCTTCCCTGTCTCTCAAATGGCTTCCCTGACGCAAACTTT
		mCCNCC	CAGAACTCATATGTCAAATAAATTTTGTTGAAGTTATCATAATAATTTTATAATATAAATGTGTTAACAAACTATATCA<br>TTAATAATATTTTAAGATAATTTTTAGTTATAATTCAGTAAGATCCTCGTATTATTTATTGTAAATTAATAAGCCCTTT<br>ACATATTAAATAACTAATGGTTGTCAGGTGCACCGAACAAAGAATAAGAAGATCCAGATTCGAGGGACATGATCT<br>CGGAGAGGCAAAGCGAACAAACGGCTACTaTaaTaaCACTTGCTATCCTCGAAATTAACGAAACGTAGTTGAAGAA<br>CCCACAGAATCAGAACCCCAACACTGCCTTATGCTTTCCCTATAAATACACATTGTCCCTTCCTCTCGTCATTTCA<br>AATACAAACTCACAACACACTTTGTACACTCCGTCCCTCTTCCCTGTCTCTCAAATGGCTTCCCTGACGCAAACTT
		mAWmCCNCC	CAGAACTCATATGTCAAATAAATTTTGTTGAAGTTATCATAATAATTTTATAATATAAATGTGTTAACAAACTATATCA<br>TTAATAATATTTTAAGATAATTTTTAGTTATAATTCAGTAAGATCCTCGTATTATTTATTGTAAATTAATAAGCCCTTT<br>ACATATTAAATAACTAATGGTTGTCAGGTGCACCGAACAAAGAATAAGAAGATCCAGATTCGAGGGACATGATCT<br>CGGAGAGGCAAAGCGAACAAACGGCTACTaTaaTaaCACTTGCTATaCaCaAAATTAAaaAAACGTAGTTGAAGAAC<br>CCACAGAATCAGAACCCCAACACTGCCTTATGCTTTCCCTATAAATACACATTGTCCCTTCCTCTCGTCATTTCAA<br>ATACAAACTCACAACACAaTaTaTACACTCaaTCCCTCTTCCCTGTCTCTCAAATGGCTTCCCTGACGCAAACTTTC
**Glyma.13G057400**	***BCCP2***	WT	GCACGTTATCGTTAAGTCAAAAGTGTACTCTTCTTTTGTACATACCAATGATGATACAATACCATATTGGAAAAGTC<br>AAAGACCTACAAAATCGGCCACGATAATAGCAAAAGCAGTGGCCATAGATCGATCAGTGCACTTGGAAGCTGTTT<br>CGATCGTTGAAGACACCAACGTTGTGCACTTGGCTCTAGAGGGAAACATCAAACATGTCTCCGAGACAAATGCAA<br>AATCAGTGACTACAAGCCACGACATATGGTTTTTATTGTTGTTTGTTGCAAGTTAGTTAAAACTGTGTCACATTGAT<br>GGTTATTGCACCACTTGCATCGTAATCATCGAAGCCACACTTGTAAAAATCTAAACTAAAACCAAACCAAACCAAA<br>CTAAAGTGTTCCAATTCTTCTTTGTATTCTCCATCCACCCTCATTGCACTTTTTAGTTTTTACCCTTTTTCGGTGTCT
		mAW	GCACGTTATCGTTAAGTCAAAAGTGTACTCTTCTTTTGTACATACCAATGATGATACAATACCATATTGGAAAAGTC<br>AAAGACCTACAAAATCGGCCACGATAATAGCAAAAGCAGTGGCCATAGATCGATCAGTGCACTTGGAAGCTGTTT<br>CGATCGTTGAAGACACCAACGTTGTGCACTTGGCTCTAGAGGGAAACATCAAACATGTCTCCGAGACAAATGCAA<br>AATCAGTGACTACAAGCCACGACATATGGTTTTTATTGTTGTTTGTTGCAAGTTAGTTAAAACTGTGTCACATTGAT<br>GGTTATTGCACCACTTGaAaaaTAATCATaatAaCCACACTTGTAAAAATCTAAACTAAAACCAAACCAAACCAAACT<br>AAAGTGTTCCAATTCTTCTTTGTATTCTCCATCCACCCTCATTGCACTTTTTAGTTTTTACCCTTTTTCGGTGTCTTC
**Glyma.05G221100**	***CAC2***	WT	GGTCGAAAGAAACCCAATTGTGGTGGGCCACGTAGCAGTCCAAATTGTCCGAGTACCTTCCAACTAGATTTAGAT<br>TTAGGCGGAGCTTTCAATTTCGCACCTCCCTCGTTTCCATCGACATCCGCATCAAAAGACCTTCTCTTTCTCTTGC<br>CATCATTTCATTCCCCATACGCTCCATCCATCACCCTTTCTTTCATTCTG
		mAW	GGTCGAAAGAAACCCAATTGTGGTGGGCCACGTAGCAGTCCAAATTGTCCGAGTACCTTCCAACTAGATTTAGAT<br>TTAGGCGGAGCTTTCAATTTCGCACCTCaCaCaTTTCCATaaACATCCGCATCAAAAGACCTTCTCTTTCTCTTGCC<br>ATCATTTCATTCCCCATACGCTCCATCCATCACCCTTTCTTTCATTCTG
		mCCNCC	GGTCGAAAGAAACCCAATTGTGGTGGGCCACGTAGCAGTCCAAATTGTCCGAGTACCTTCCAACTAGATTTAGAT<br>TTAttCttAtCTTTCAATTTCGaAaaTtCCTCGTTTCCATCGACATCCGCATCAAAAGACCTTCTCTTTCTCTTGCCATC
		mCCNCCv2	GGTCGAAAGAAACCCAATTGTGGTGGGCCACGTAGCAGTCCAAATTGTCCGAGTACCTTCCAACTAGATTTAGAT<br>TTAttCttAtCTTTCAATTTCGCACCTCCCTCGTTTCCATCGACATCCGCATCAAAAGACCTTCTCTTTCTCTTGCCAT<br>CATTTCATTCCCCATACGCTCCATCCATCACCCTTTCTTTCATTCTG
		mAWmCCNCC	GGTCGAAAGAAACCCAATTGTGGTGGGCCACGTAGCAGTCCAAATTGTCCGAGTACCTTCCAACTAGATTTAGAT<br>TTAttCttAtCTTTCAATTTCGaAaaTaaCaCaTTTCCATaaACATCCGCATCAAAAGACCTTCTCTTTCTCTTGCCATCA

Note: Mutated sequences are shown as lower-case letters.

AW BOX

CNC BOX

Table S2. Primers Used to Clone GmWRI1-A and WRI1 Binding Sites

locus	ID	Forward primer *	Reverse primer *	Destination Vector
Glyma.08G227700	GmWRI1-A	TACAATTACAGTCGA ATGAAGAGGTCTCCAGCATCTTCTTG	TGCAGCCGGGCGGCC TCATAGATCTAGAGCATAGTCACAAGAAACTG	CaMV35S- sGFP(S65T)-NOS plasmid digested with SalI and NotI
Glyma.19G240100	ACP3-WT	GCTCTAGAGGACTAG GATTGTGGATTTCATTATACTAC	GAAGGGTCTTGACTAG GGTCGTGAGGAGTAAAAG	pDLUC15 plasmid digested with SpeI
Glyma.15G098500	ACP4-WT	GCTCTAGAGGACTAG CAGAACTCATATGTCAAATAAATTTTGTTG	GAAGGGTCTTGACTAG GGAACAGAAGCAAGTGAAATGAAG	pDLUC15 plasmid digested with SpeI
Glyma.13G057400	BCCP2-WT	GCTCTAGAGGACTAG GCACGTTATCGTTAAGTCAAAAGTG	GAAGGGTCTTGACTAG GGGGTGCGTGATCTGCAAAG	pDLUC15 plasmid digested with SpeI
Glyma.05G221100	CAC2-WT	GCTCTAGAGGACTAG CGTATTTAACCATTAATGTTGC	GAAGGGTCTTGACTAG CTATGGCTTTACCTACGCT	pDLUC15 plasmid digested with SpeI

***Note:** The sequences TACAATTACAGTCGA and TGCAGCCGGGCGGCC were added to primers F and R, respectively. These sequences correspond to multisite cloning sequences for the insertion of the WRI1 cDNA into the CaMV35S- sGFP(S65T)-NOS plasmid

***Note2:** The sequences GCTCTAGAGGACTAG and GAAGGGTCTTGACTAG were added to primers F and R, respectively. These sequences correspond to overlapping sequences to the pDLUC15 vector that were required for in-fusion cloning.
